# Advantages of Intranasal Vaccination and Considerations on Device Selection

**Published:** 2009

**Authors:** M. Birkhoff, M. Leitz, D. Marx

**Affiliations:** Pharma Division, ING. ERICH PFEIFFER GMBH, Öschlestrasse 54-56, 78315 Radolfzell, Germany; 1 Pharma Division, ING. ERICH PFEIFFER GMBH, Öschlestrasse 54-56, 78315 Radolfzell, Germany; 2 Pharma Division, ING. ERICH PFEIFFER GMBH, Öschlestrasse 54-56, 78315 Radolfzell, Germany

**Keywords:** Vaccination, intranasal vaccines, nasal spray, dry powder delivery systems

## Abstract

Oral and Intramuscular vaccination has been considered till date as the ultimate ways, but nasal route offers advantages such as ease of self administration and induction of mucosal as well as systemic immunity. Both liquid and dry powder formulations can be given via intranasal route. A great consideration has to be given while selecting a suitable device for nasal administration since the volume delivered is very low. A number of devices are available based on number of doses to be administered and type of dosage formulation.

In the past, the intramuscular and oral administrations of vaccines were considered the ultimate ways. Intranasal vaccination is a viable alternative, because it most often resembles better the natural way of infections, self administration is easily done and therefore may gain a reasonable share within the next couple of years.

## ADVANTAGES OF INTRANASAL VACCINATION

For most microbes, the nasal mucosa is the first barrier which must be conquered. So it's not a surprise, that this mucosa is very immune-competent. It was shown, that even small amounts of antigen elicit a protective response. This will become a striking argument, if the poor yields with the current H1N1 vaccine seed strains and the workload linked to virus production in eggs are considered. It is also a clear advantage, that nasal vaccination induces both mucosal (protection at site of infection) and systemic immunity. In contrast, intramuscular vaccination primarily induces systemic immune response (antibody formation). In addition, intranasal vaccination may confer protection against infections at other mucosal sites, such as the lungs, intestines and genital tract, and provide cross-protection against variant strains through mucosal antibody secretion. Another important advantage: the nasal cavity is easily accessible.

Intranasal drop or spray administration is not invasive and causes little discomfort to patients. This is important, because many people fear injections because they are linked to pain, disease transmission (HIV or hepatitis B) and an anaphylactic response may happen.

Intranasal administration may be best suited for barrier vaccinations, following the outbreak of highly infectious diseases, because less skilled persons like pharmacists or nurses can do mass vaccinations.

Intranasal vaccines may be most beneficial for special populations:

- children (easy to use, non-invasive)
- elderly patients (easy to use, non-invasive)
- HIV-infected patients (no fear for needle stick injuries)
- multi-morbid patients (fed up with injections)

## COST DRIVING FACTORS FOR VACCINATION

Up to now, quite all approved vaccines are liquids for oral or intramuscular administration. At least in countries with well established infrastructure, vaccines are delivered as single dose prefilled syringes or vials. In other countries, much cheaper multidose bottles are the mainstay, which may be also used for intranasal vaccines. The price for a vaccine, its primary packaging and the delivery device is not equal to the cost for vaccination. Depending on temperature-sensitivity and required space, the storage and transportation in the cold-chain can cause substantial costs. A quite high percentage of vaccines have to be discarded due to failure in the cold-chain (so called “wastage”). Looking on how fast vaccination could be provided in pandemic situations, it is important, who can do a save administration. It makes a difference, if a physician have to do it (injections), or nurses or pharmacist can handle it. For intranasal vaccines, even self-administration is a save option.

A clear advantage for the intranasal route is, that liquids and dry powder formulations may be used. The latter should provide clear advantages on the transportation and wastage issues, because a cold-chain may not be required and a longer shelf live should be achieved.

## CONSIDERATIONS ON DEVICE SELECTION

When selecting a device for nasal administration, it must be considered, that the administration volume is comparable low. For liquids, a volume of 100 μl is optimum per nostril in adults, but should be reduced for children to avoid nasal dripping. A device with high spray performance will reduce the amount of antigen, needed to elicit reliable protection. It is a general decision, if the vaccine shall be administered in one or both nostrils. The latter method seems to give patients more confidence and will increase acceptance for that route. The immediate packaging of the vaccine (dry powder or liquid) must be optimized for easy, automated filling (tiny amounts and large quantities) and must provide reliable protection for storage and transportation.

Single dose devices will give best protection for the vaccine, but require highly sophisticated filling technology. Because these systems are quite expensive and bulky, it is only suited for countries with a well developed infrastructure. Multi-dose spray pumps are an option for liquid vaccines, if an inuse microbial contamination of the bottle content can be prevented. So called “preservative free pump systems” can fulfill this requirement and are very cost effective. Transmission of diseases from patient to patient can be effectively prevented using disposable sleeves or protection caps.

### Unitdose liquid (fig. 1)

**Fig. 1:**
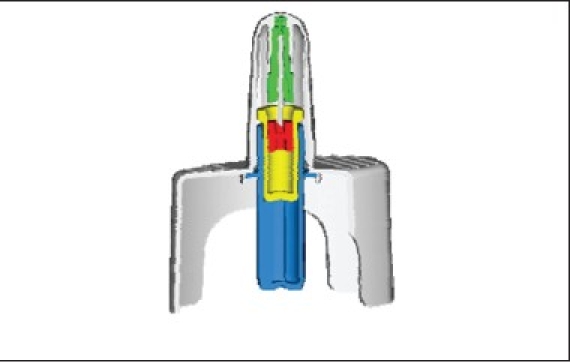
Unitdose nasal liquid spray system

- Single dose nasal spray system
- Hermetically sealed primary glass container
- Dose volume: 100 μl

### Bidose liquid (fig. 2)

**Fig. 2:**
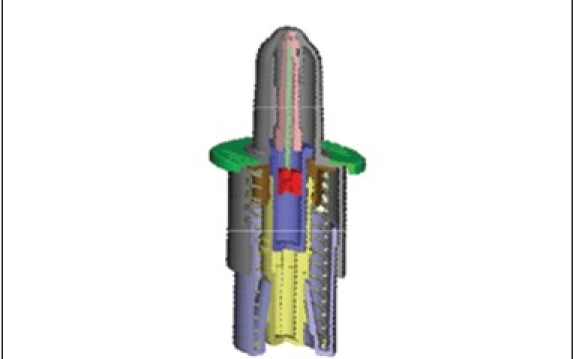
Bidose nasal liquid spray system

- Bidose nasal spray system
- Hermetically sealed primary glass container
- Dose volume: 2 × 100 μl

### Multi-dose liquid device with disposable sleeve/protection cap (fig. 3)

**Fig. 3:**
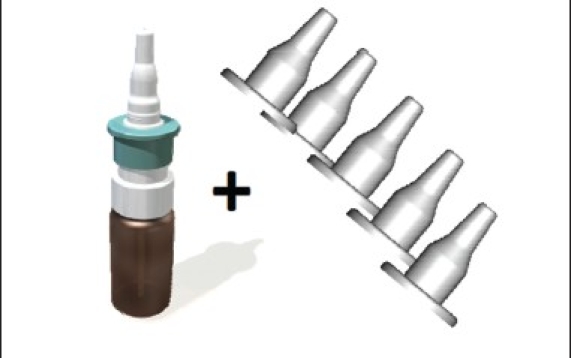
Multidose liquid device

- Multi-dose pump with tip-seal technology prevents contamination of bottle content
- snapped on standard glass bottles 5-20 ml
- 70-**100**-140 μl per actuation
- single use sleeve/protection cap for each patient prevents disease transmission

### Unitdose powder (fig. 4)

**Fig. 4:**
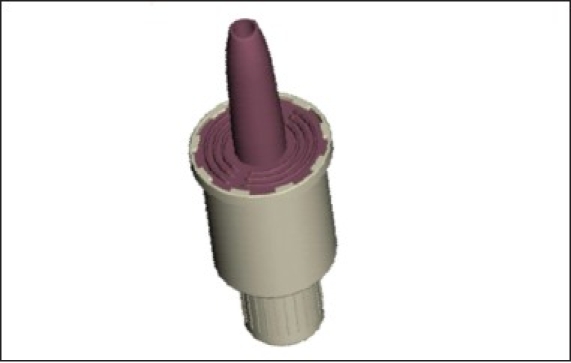
Unitdose nasal powder delivery system

- Active, single dose nasal powder delivery system
- No need to coordinate actuation with inhalation
- Max filling volume: 140 mm^3^ (20-50 mg)
- Conventional filling technology (capsule-type)
- In combination with a spacer suited for inhaled vaccines

### Bidose powder (fig. 5)

**Fig. 5:**
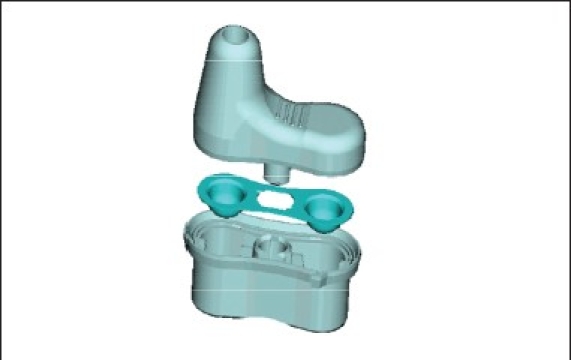
Bidose nasal powder delivery system

- Bidose nasal powder delivery system
- Passive technology
- Best protection of the powder formulation due to special blister laminate (foil)
- Max filling volume: 190 mm^3^ (50-100 mg) per chamber
- Intranasal vaccines may save costs for massvaccinations, because much less antigen is needed and it is a save and easy administration route
- Devices for liquid or dry powder administration are available, which are compatible with automated filling and assembly technology
- For liquid vaccines (e.g. pandemic influenza) costeffective and save multi-dose solutions can be used
- The non-invasive administration and the potential use of dry-powder formulations may further assist its wider use

